# Consensus between Pipelines in Structural Brain Networks

**DOI:** 10.1371/journal.pone.0111262

**Published:** 2014-10-30

**Authors:** Christopher S. Parker, Fani Deligianni, M. Jorge Cardoso, Pankaj Daga, Marc Modat, Michael Dayan, Chris A. Clark, Sebastien Ourselin, Jonathan D. Clayden

**Affiliations:** 1 Centre for Medical Image Computing, University College London, London, United Kingdom; 2 Imaging and Biophysics Unit, UCL Institute of Child Health, London, United Kingdom; 3 Dementia Research Centre, University College London, London, United Kingdom; 4 Department of Radiology, Weill Cornell Medical College, New York, New York, United States of America; Chinese Academy of Sciences & National Laboratory of Pattern Recognition, China

## Abstract

Structural brain networks may be reconstructed from diffusion MRI tractography data and have great potential to further our understanding of the topological organisation of brain structure in health and disease. Network reconstruction is complex and involves a series of processesing methods including anatomical parcellation, registration, fiber orientation estimation and whole-brain fiber tractography. Methodological choices at each stage can affect the anatomical accuracy and graph theoretical properties of the reconstructed networks, meaning applying different combinations in a network reconstruction pipeline may produce substantially different networks. Furthermore, the choice of which connections are considered important is unclear. In this study, we assessed the similarity between structural networks obtained using two independent state-of-the-art reconstruction pipelines. We aimed to quantify network similarity and identify the core connections emerging most robustly in both pipelines. Similarity of network connections was compared between pipelines employing different atlases by merging parcels to a common and equivalent node scale. We found a high agreement between the networks across a range of fiber density thresholds. In addition, we identified a robust core of highly connected regions coinciding with a peak in similarity across network density thresholds, and replicated these results with atlases at different node scales. The binary network properties of these core connections were similar between pipelines but showed some differences in atlases across node scales. This study demonstrates the utility of applying multiple structural network reconstrution pipelines to diffusion data in order to identify the most important connections for further study.

## Introduction

Studying brain structural networks using diffusion MRI tractography has recently become a popular research topic in neuroscience [1]. In this field the research aim is to quantify connectivity between grey matter regions via white matter pathways *in vivo*, on a global scale, in order to understand the topological organisation of brain structure and to relate this to aspects of neurological health and disease. The structural topology may be analysed by characterising the brain as a graph, whereby sets of network nodes, representing grey matter regions, transfer information between one another via network edges, representing connecting axonal pathways.

It has been suggested that the organisation of the structural network may reflect neurological phenotype. For example, network metrics such as clustering coefficient and pathlength have been related to the effect of age [2], gender [3] and IQ [4]. In addition, network alterations have been observed in neurological diseases such as Alzheimer's disease [5], [6], epilepsy [7], [8] and schizophrenia [9]–[11], meaning such metrics may become useful as topological biomarkers of brain integrity or pathology. However, reconstructing brain network nodes and edges is both a conceptual and practical challenge and there is little agreement between studies of how exactly these should be defined.

Nodes of the brain network, which represent spatially distinct regions of grey matter, may be defined using different parcellation schemes and scales. A common parcellation technique has been to warp the structural image to an anatomical template, such as the AAL atlas [12], where the grey matter regions have been manually labelled in a single representative subject [4], [9], [11], [13]. Alternative warping strategies have been applied; for example, those utilising cortical shape and curvature information [14], or multiple template propagations [15]. In addition, different templates may be used to generate different parcellation schemes. Most parcellations used in whole-brain structural network studies have been relatively coarse, with around 100 brain regions. Because of the uncertainty concerning where to place region boundaries, some studies have divided parcellated regions into smaller pseuodorandom patches [16], [17], or performed network analysis across a range of parcellation scales [18]–[20]. Regions defined in structural space must then be accurately warped to diffusion space in order to estimate the inter-regional connectivity, and a number of registration schemes are available.

Edges of the structural brain network, which represent white matter tracts between two grey matter regions, are frequently quantified based on the number of connecting fibers. As such, the issue of which fiber model, initialisation and tracking technique to use arises. The diffusion tensor model [21], combined with deterministic tracking, is a common technique for reconstructing network edges [14], [22]–[24]. Multiple fibers may be represented using multiple diffusion tensors, the orientation distribution function (ODF) [25], multi-compartment models [26] and the fiber orientation distribution (FOD) [27]. The ball and sticks multi-compartment model is one example of a popular fiber model employed to track through multiple fiber populations in structural network studies [3], [28]. In contrast to multi-compartment models, the FOD representation assumes an identical signal response for each fiber population and does not employ model fitting. Deterministic tracking determines inter-regional connectivity by following the dominant fiber orientation whereas probabilistic tracking samples directions from a distribution of orientations to produce a connectivity distribution.

In networks obtained using probabilistic tractography, a continuous measure of connectivity is generated which reflects (to some degree) the probability of connection between all brain regions. A probability threshold may then be applied to produce a binary network, where connections are either absent or present [29]. However, assigning importance to connections is a challenge and the choice of threshold affects the occurance of false positive and negative connections, resulting in a trade-off between sensitivity and specificity of the connections [30], [31]. Thresholding also has an intrinsic impact on the network topological measures [32].

Given the complexity and number of steps involved in network reconstruction from the raw diffusion MRI images, it is important to provide an assessment of consensus in networks obtained from alternative reconstruction pipelines which vary not in just one or two components but in the entire reconstruction pipeline (i.e the parcellation, registration and fiber model). This would also enable some assessment of the potential impact in swapping and substituting individual components of the reconstruction.

In this study, we assessed the convergence of structural connectivity networks obtained from two alternative pipelines across a range of network density thresholds, by merging alternative parcellations to a common and equivalent node scale. This allowed us to investigate similarity between independent network reconstructions on a connection-wise basis and to identify the underlying brain connections occuring most robustly in both pipelines. Our results suggest it may be useful to apply multiple pipelines to obtain structural brain networks from diffusion data and to employ the comparison framework described here to identify the most important connections.

## Methods

### Ethics statement

Informed written consent was obtained from all subjects participating in this study. Processes for consenting and image acquisition were approved by the UCL Research Ethics Committee.

### Subjects and image acquisition

Twenty-eight young healthy adult subjects (16 male, mean age 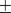 s.d. 28.5 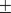 3.9 years) participated in this study. Subjects had no brain abnormalities at the time of scanning, as determined by examination of their structural scan by an expert radiologist. Two T1-weighted images of 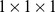 mm resolution were acquired sequentially with a 3D Fast Low-Angle Shot (FLASH) sequence (176 contiguous sagittal slices, 256×224 mm FOV, TR = 11 ms, TE = 4.94 ms and 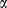  = 15°) on a 1.5T Siemens Avanto MRI scanner at Great Ormond Street Hospital, London. A diffusion-weighted echo planar sequence (TR  = 7300 ms, TE = 81 ms) with 60 noncollinear diffusion directions (b = 1000 s/mm^2^) was used to acquire diffusion-weighted images of 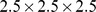 mm and three un-weighted images (b = 0 images). The diffusion-weighted sequence was repeated three times for each subject in a single scanning session.

### Image preprocessing

DICOM images were converted into NIfTI format using TractoR [33] and the brain was extracted from all images using FSL's brain extraction tool [34]. In order to increase the signal to noise ratio of the structural image, the two acquired T1-weighted images were registered and averaged in Freesurfer v5.1.0 [35]. The diffusion-weighted volumes were corrected for eddy-current induced distortions by affine registration to an unweighted reference image using the diffusion-specific FSL FDT algorithm [36].

**Figure 1:**
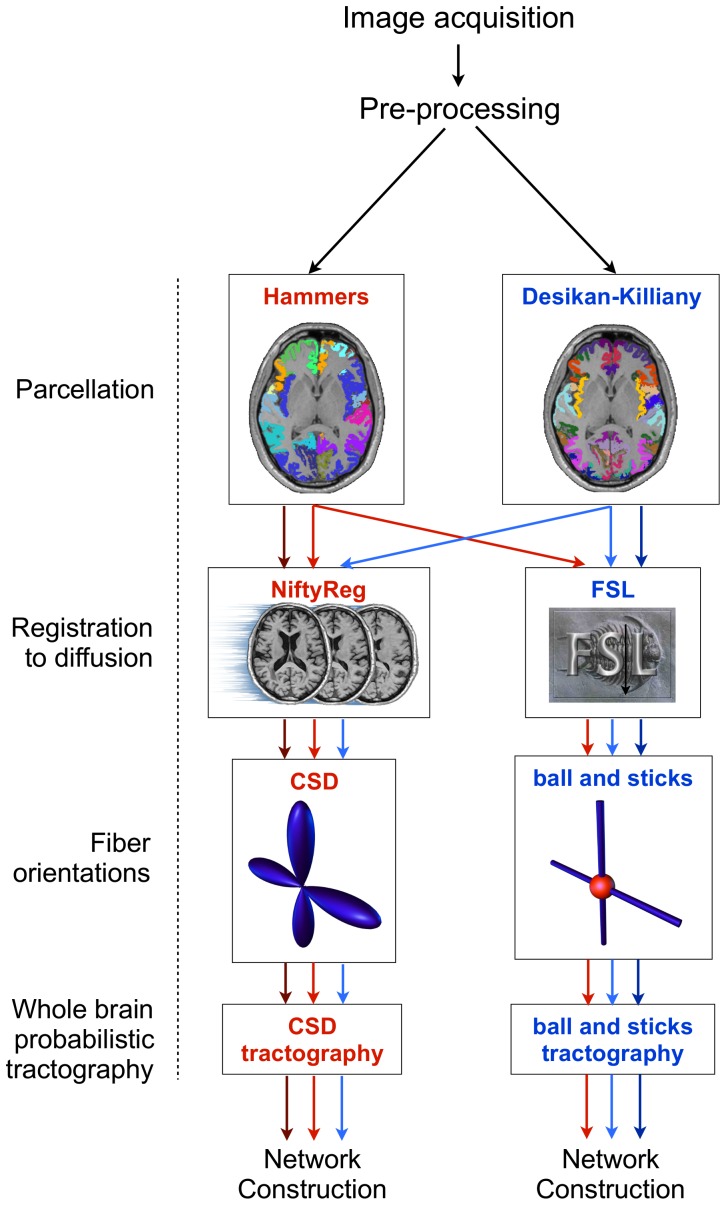
Summary of network reconstruction stages applied to structural and diffusion images for P1 and P2. The pipeline stages are shown on the left and the alternative implementations of the methods are shown inside the boxes. Arrows indicate the passage of merged (dark arrows) and native (light arrows) atlases through the pipeline stages (red and blue refer to Hammers and Desikan-Killiany atlases, respectively). Nodes were defined by registration of the cortical parcels to diffusion space. Edges were defined by performing tractography from the parcel boundary through the fiber orientations. Note that the whole-brain probabilistic tractography methods differed only in relation to the recommended settings for the software used to track through the fiber orientations. The network construction stage calculated the connecting fiber density between all cortical parcel pairs across the entire cerebral cortex and was identical for both pipelines. Applying these stagesto the merged and native atlases resulted in comparisons between pipelines at three node scales; the merged atlas scale (34 nodes, dark arrows), Hammers atlas scale (44 nodes, light red arrows) and Desikan-Killiany scale (68 nodes, light blue arrows). We also applied the registration and whole-brain tractography pipelines to the AAL atlas (not shown).

We chose to compare two alternative state-of-the-art reconstruction pipelines (these two pipelines will hereafter be referred to as P1 and P2, Fig 1). Both reconstructions had similar capabilities but varied with respect to the details of the cortical parcellation, registration and probabilistic fiber model method.

### Cortical parcellation

To define network nodes, the cortical grey matter of the averaged T1-weighted image was parcellated into regions using automated software. **P1**. NiftySeg was used to parcellate the structural image into 44 cortical regions (22 per hemisphere), as defined by the Hammers Atlas [37]. The parcellation algorithm first labels brain regions by propagating a set of manually labelled T1-weighted images to the structural image [38], [39]. The LoAd tissue segmentation algorithm was then applied to the structural image to obtain the cortical grey matter of the parcellated regions [40]. **P2**. Freesurfer was used to parcellate the structural image into 68 cortical regions (34 per hemisphere), as defined by the Desikan-Killiany Atlas [41]. The parcellation algorithm assigns a neuroanatomical label to each location on a cortical surface model of the image, based on probabilistic information from a manually labeled training set [42].

### Native and common node scale parcellations

**Figure 2:**
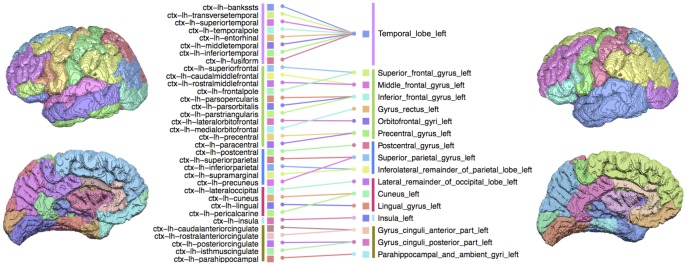
Merging cortical parcels of P2 parcellations. The native scale P2 parcellation (68 parcels) is shown on the left and the merged P2 parcellation (34 parcels) is shown on the right. The merging pattern was identical for both hemispheres and therefore only the left hemisphere is shown. The colour scheme of brain regions is as in Fig. 2. Lines represent merging of native scale parcels (left) to their equivalent common scale parcels (right). Coloured vertical lines correspond to regions in the temporal (purple), frontal (green), parietal (blue), occipital (red), insula (light-blue) or limbic (yellow) lobes. Native scale P1 parcellations (44 nodes) were merged to the common scale parcellation by merging all temporal lobe parcels.

**Figure 3:**
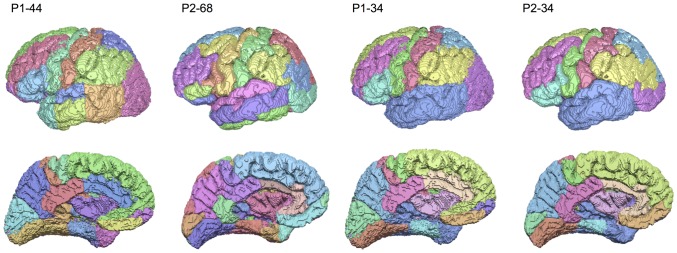
Representative cortical parcellations of P1 and P2 at the native and common node scale. Temporal lobe regions in P1 native scale parcellations (P1-44, far left) were merged, resulting in a lower scale parcellation (P1-34, middle right). Selected regions across the entire cerebral cortex in P2 native scale parcellations (P2-68, middle left) were merged (P2-34, see Fig. 1). This resulted in a common and anatomically equivalent parcellation scale of 34 nodes for both P1 and P2 networks.

The pipelines employed atlases with a different number of brain regions, preventing a direct connection-wise comparison between them. Therefore, parcels in the native atlases were merged to a common node scale (Fig. 2). The number of merges was the minimum required to give correspondence between the atlases and resulted in 34 brain regions (17 per hemisphere). Parcels in the Desikan-Killiany atlas (P2) were merged across the entire cortex based on anatomical correspondence to their equivalent Hammers atlas (P1) parcels. For example, the pars opercularis, pars orbitalis and pars triangularis parcels in the native Desikan-Killiany atlas corresponded to the inferior frontal gyrus parcel in the native Hammers atlas and therefore in both of the merged atlases. The Desikan-Killiany and Hammers atlases differed fundamentally in temporal lobe regions, meaning an equivalent merging of parcels could not be found. Therefore, the temporal lobe is itself considered as a single node in both merged parcellations (Fig. 2 and 3). The merging process did not result in identical parcellations. The remaining differences in common scale parcellations were due to alternative border criteria as well as alternative parcellation algorithms. Therefore, in addition to the native Desikan-Killiany and Hammers atlases, we also obtained the two merged 34 node scale versions of each atlas for each subject (Fig. 3).

Each common scale parcellation was registered to diffusion space (as described below) using the registration implementation for the corresponding pipeline (e.g. following the P1 registration-tractography for the merged P1 parcellation). We also applied the paired registration-tractography implementation from each pipeline to both of the native atlases. We further tested the robustness of our results by applying each registration-tractography implementation to the Automated Anatomical Labelling (AAL) Atlas parcellation [12], which had cortical 78 regions.

### Registration of cortical parcels to diffusion space

The structural and diffusion-weighted images were co-registered in order to define the cortical parcels of interest in diffusion space. The registration field was determined as follows. An affine registration was used to register the first b = 0 image to the averaged T1-weighted image. The T1-weighted image was then non-linearly registered to the b = 0 image using the inverse of the transformation acquired in the previous stage as a starting transformation. The transformation field was retained and applied to the cortical parcellation to transform parcels to diffusion space. The categorical nature of the labels was preserved through a nearest neighbour resampling scheme. **P1**. NiftyReg was used to perform the linear and non-linear registrations using the default settings [39], [43]. NiftyReg used normalised mutual information to calculate image similarity and a bending energy regularisation with cubic B-spline parameterisation for the non-linear warping. **P2**. The linear and non-linear registration was performed by FSL FLIRT and FNIRT, respectively [36]. Normalised cross correlation and sum-of-squared difference was used to calculate image similarity for the linear and non-linear warping stages, respectively. The membrane energy was used to regulate the non-linear warp field which was parameterised as a cubic B-spline scheme.

### Fiber orientations

The orientations of fiber bundles at each voxel were inferred using one of two methods. **P1**. Constrained spherical deconvolution (CSD) was applied to estimate the underlying fibre orientation distributions (FOD) in each voxel, using MRTrix [44]. CSD assumes that the observed diffusion signal is a convolution of fiber orientations and a diffusion signal response function, meaning the fiber orientations may be extracted by spherical deconvolution of the diffusion signal. The maximum spherical harmonic order for the deconvolution was set to 8. **P2**. A ball and two sticks multi-compartment fiber model was fitted to the diffusion data, using the Bayesian Estimation of Diffusion Parameters Obtained using Sampling Techniques (BEDPOSTX) algorithm in FSL. The BEDPOSTX algorithm uses Markov chain Monte Carlo sampling to estimate the uncertainty in fibre orientations [45].

### Probabilistic fiber tractography

The paths of fiber trajectories in the brain were reconstructed by seeding 100 probabilistic fibers from the interior boundary voxels of each cortical parcel. The interior boundary voxels were the intersection of the dilated binary cortical parcellation with the fiber propagation mask (defined for each pipeline below). **P1**. Fibers were propagated using the default settings in MRTrix [46]. The sampling interval was 0.2 mm, maximum curvature threshold was 60° and minimum fiber orientation dispersion (FOD) amplitude threshold for tracking through a voxel was 0.1. The propagation mask was defined as the union of white matter, sub-cortical grey matter and ventricle regions from the LoAd tissue segmentation provided by NiftySeg. **P2**. The default settings in FSL ProbTrack algorithm were used to determine the fiber trajectories [26]. The sampling interval was 0.5 mm and stopping criteria meant that fibers terminate if they curve by more than 80°. The propagation mask was defined as the white matter segmentation provided as part of the Freesurfer output, and included white matter, sub-cortical and ventricular regions.

Note that P1 initiates fibers by uniform sampling of boundary voxels with a FOD amplitude greater or equal to 0.2, whereas P2 initiates fibers from the centre of each boundary voxel. Also, P1 terminates fibers if the FOD amplitude is below 0.1. For both pipeline tracking schemes, fibers were terminated immediately after leaving the propagation mask so that their cortical parcel connections could be recorded.

### Network construction

Network construction and analysis was performed using the R programming language [47]. Cortical parcels were represented as network nodes and the fiber connections between them as edges. Fibers connected node pairs if their end-point coordinates terminated within two distinct cortical parcels. The connection weight between two cortical nodes was defined as the density of connecting fibres (as in [48]), calculated as the sum of connecting fibers divided by the mean volume of the seed (boundary) voxels adjacent to the two parcels (boundary voxels were assigned to the nearest parcel by Euclidean distance). Performing this calculation for all fibers produces an *N*-by-*N* undirected matrix of connection weights, where 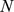 is the number of nodes in the parcellation (either 34, 44, 68 or 78). The weighted cortical connection matrix was calculated for the repeat diffusion scans of all subjects. The subject mean weighted connection matrices (across the three repeat diffusion scans) were calculated for all subjects by averaging each weight across all scans.

### Convergence between alternative pipelines

Convergence between alternative pipelines was investigated in binary networks of equal density. Binary networks were generated by thresholding the subject weighted networks and convergence was quantified for all possible densities in the range [0,1], by selecting the 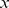 highest ranked connections in the weighted matrix, for 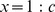, where 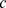 is the total number of possible connections (calculated as 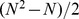). Connections of equal weight (predominantly weights of value 0), were randomly assigned a rank, meaning connections were chosen randomly if the network density threshold intersected connections of equal weight. Convergence was quantified between the pair-wise subject binary networks using the Dice Similarity Coefficient (DC). DC was defined as the proportion of intersecting connections relative to the total number of connections at that density. This measure is identical to the percentage convergence measure of network similarity used in [49] for networks of equal density.

Our investigation was interested in similarity between pipelines independent of network density effects (denser networks have a higher DC by chance). Therefore, at each network density, we computed a one sample t-statistic between the observed DC across all subjects to the expected DC value, using a two-tailed t-test. The expected DC value was equal to the network density, 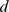, as the number of connections expected to agree in two random binary networks (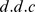) was divided by the total number of connections (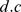). Our null hypothesis was that similarity between pipelines was equal to that by chance, given the density. The p-value computed from this t-statistic was our estimate of the significance of the similarity. To estimate the dependency of the significance on our sample population, we bootstrapped the subjects 1000 times at each network density. As lower p-values represented higher similarity, we inspected the negative logarithm of the p-value to obtain a global maximum significance and corresponding network density where the binary network similarity was most reliably different from random.

### Network properties of the consensus network

The binary networks corresponding to the peak convergence threshold will hereafter be referred to as ‘consensus networks’ for convenience. The graph theoretical proprerties of the consensus networks were calculated for all subjects using the igraph package [50] in the R programming language. The global properties of characteristic pathlength [51] and global efficiency [52], and the local properties of local efficiency [52], clustering coefficient [53] and assortativity [54], were calculated as described in [55].

## Results

### Convergence between alternative network reconstructions

The raw weights matrix represents the connecting fiber density between cortical region pairs across the entire cerebral cortex. The connecting fiber density was highly correlated between subject mean networks obtained from alternative reconstructions in terms of both rank and weight (34 nodes: Spearman 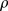  = 0.675 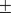 0.06, Pearson 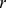  = 0.630 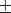 0.061, 44 nodes: Spearman 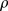  = 0.677 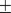 0.076, Pearson 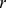  = 0.702 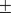 0.085, 68 nodes: Spearman 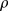  = 0.586 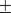 0.095, Pearson 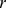  = 0.632 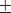 0.085), confirming that these pipelines had yeilded similar networks.

A general trend of decreasing DC with decreasing network density was observed. The grand mean DC across all subjects and densities was 0.741 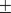 0.165, 0.759 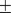 0.132 and 0.724 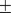 0.135 for the 34, 44 and 68 atlas scales, respectively. At a network density of 1 the DC was 1 as all connections existed in both pipelines. Clearly, the DC should be interpreted in the context of the expected similarity of random networks at the same density (Fig. S1).

**Figure 4:**
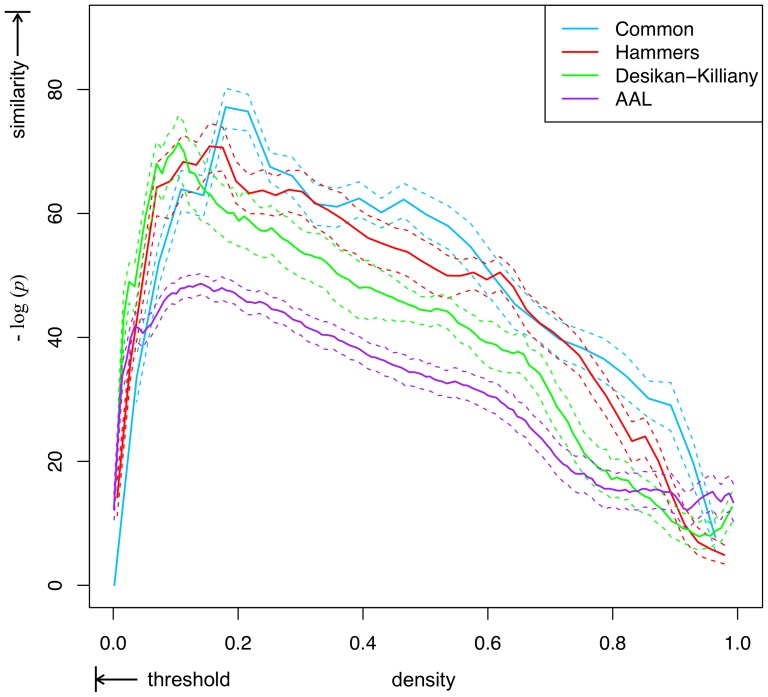
Similarity between P1 and P2 networks across density thresholds using atlases at three node scales. Significance of similarity was calculated by comparing the distribution of within-subject DC to the expected DC by chance, given the density of the networks. Shown is the mean negative 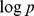-value of the DC between binary networks thresholded at a given density. The within-subject DC's were bootstrapped to obtain a standard error on the mean (dashed lines). A global peak similarity was found at a density of 0.196, 0.161, 0.106 and 0.142 for the Common (34 nodes), Hammers (44 nodes), Desikan-Killiany (68 nodes) and AAL (78 nodes) atlases, respectively.

The networks were significantly more similar between pipelines than by chance across all density thresholds (Fig. 4). Similarity increased approximately linearly with increasing threshold (corresponding to a decreasing network density), until very high thresholds were reached, where a peak similarity was observed (at network densities between 0.1–0.2, depending on the atlas), after which similarity decreased sharply towards 0. The peak similarity threshold resulted in binary networks that were most highly similar between the pipelines whilst accounting for the expected similarity at this density by chance. The most highly significant similarity was found at densities of 0.196, 0.161 and 0.106 (110, 152 and 242 connections) for node scales of 34, 44 and 68, respectively. The magnitude of the significance was similar between atlases of different scales (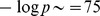) at the peak similarity threshold.

Similar results were obtained using the AAL atlas. The weighted networks were highly correlated between pipelines in terms of rank and weight (Spearman 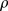  = 0.703 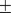 0.161, Pearson 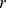  = 0.692 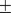 0.162) and the grand mean DC was 0.701 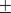 0.114 (Fig. S1). The peak similarity was observed at a density of 0.142 (427 connections), where 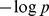 was 48.9 (Fig. 4).

**Figure 5:**
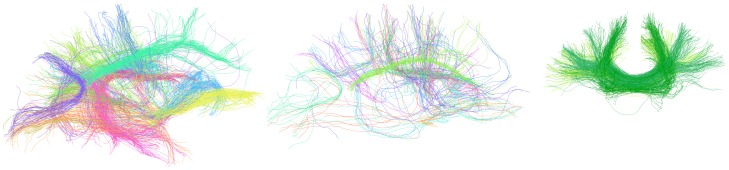
P1 fibers underlying convergent connections in the left hemisphere of a repre-sentative subject. Fibers are coloured by their network connection. (a) Inter-lobe fibers viewed from the medial aspect. (b) Intra-lobe fibers viewed from the medial aspect. (c) Inter-hemispheric fibers shown from the coronal aspect. The paths of fibers underlying convergent inter-lobe connections agrees with that of major anatomical tracts, such as the ILF (orange) and cingulum (green). Convergent intra-lobular connections were mostly represented by short-range cortical U-fibers. Convergent inter-hemispheric fibers travel via the corpus callosum and connected homotopic cortical regions, such as the superior, middle and inferior frontal gyri (green). For visual clarity, a maximum of 200, 50 and 100 fibers from the subset of whole-brain tractography fibers are shown per connection for (a), (b) and (c), respectively. Also, only fibers greater than 7 cm are shown for (a) and (b) and greater than 10 cm for (c).

The paths of fibers underlying peak convergent connections are shown in Fig. 5. Fibers representing the inter-lobe connections, intra-lobe connections and inter-hemispheric connections are shown for a representative subject reconstructed through the P1 pipeline. By visual inspection, it can be appreciated that the spatial distribution of fibers corresponds with known major anatomical tracts according to previous literature [56]. Major white matter tracts, such as the inferior longitudinal fasciculus, superior longitudinal fasciculus, cingulum and arcuate, were represented by fibers underlying inter-lobe connections. On the other hand, fibers representing intra-lobe connections appeared to be mostly cortical U-fibers.

### Network properties of consensus networks

**Figure 6:**
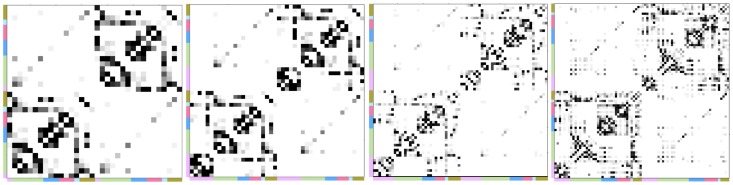
Prevalance of convergent connections across subjects at the peak convergence density. The prevalance is shown for the common (left), Hammers (middle-left), Desikan-Killiany (right-left) and AAL (right) atlases. Convergent connections were defined as the intersection of subject networks thresholded at the peak convergence density obtained from our bootstrap statistical analysis. The node lobe memberships are indicated by the adjacent colour bars, as in Figure 2. Colours represent the temporal (purple), frontal (green), parietal (blue), occipital (red), insula (turquoise) and cingulate (brown) lobes.

The convergent connections of the consensus networks are summarised in Fig. 6. The connections that agreed between pipelines tended to be similar across subjects. The convergent connections, which had high hemispheric symmetry, were primarily between ipsilateral intra-lobe regions and between bilateral homotopic regions. The left and right insula gyri were the most highly connected nodes in the consensus network.

The density of the consensus networks decreased when comparing lower to higher node scales atlases, whereas the number of connections increased. The graph theoretical metrics of global pathlength, clustering coefficient, global efficiency, local efficiency and assortativity, which were calculated for all consensus networks, are shown in Table 1. Graph theoretical properties of the consensus networks were similar between pipelines employing atlases at the same node scale, whereas some differences were found in graph theoretical properties between atlases of different node scales. The global pathlength and clustering were not significantly different between pipelines at the same scale but tended to increase and decrease in higher node scale atlases, whereas global efficiency tended to decrease. Assortativity was less stable between pipelines and showed no clear trend with node scale. The AAL atlas consensus networks had a relatively high density considering the number of network nodes in the parcellation, compared to other atlases.

**Table 1:** Graph theoretical properties of peak convergent networks.

Atlas (nodes)	DC	Connections	Density	PL	CC	G.Eff	L Eff	AS
Common (34)	0.71 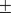 0.03	110	0.196					
P1				2.39 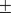 0.08	0.47 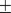 0.02	0.42 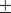 0.01	0.71 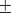 0.04	0.12 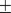 0.09
P2				2.42 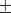 0.17	0.47 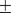 0.05	0.42 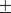 0.03	0.72 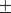 0.05	0.04 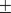 0.08
Hammers (44)	0.74 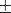 0.05	152	0.161					
P1				2.69 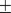 0.11	0.48 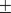 0.02	0.37 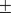 0.02	0.74 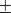 0.03	0.10 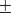 0.10
P2				2.61 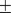 0.11	0.48 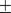 0.04	0.38 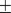 0.02	0.71 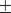 0.03	0.06 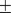 0.06
Freesurfer (68)	0.66 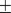 0.05	242	0.106					
P1				2.93 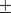 0.14	0.41 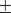 0.03	0.34 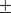 0.02	0.67 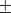 0.05	0.12 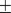 0.06
P2				2.94 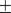 0.18	0.43 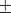 0.04	0.34 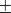 0.02	0.71 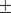 0.04	0.02 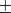 0.05
AAL (78)	0.70 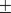 0.11	427	0.142					
P1				2.40 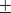 0.09	0.45 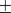 0.03	0.42 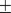 0.02	0.42 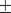 0.01	0.11 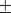 0.07
P2				2.43 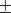 0.04	0.47 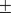 0.01	0.41 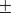 0.01	0.42 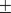 0.01	0.07 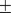 0.06

**Graph theoretical characteristics of subject binary networks thresholded at the peak convergence density.** Shown are the mean 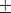 standard deviation of the graph theoretical properties of Pathlength (PL), Clustering Coefficient (CC), Global Efficiency (G. Eff), Local Efficiency (L. Eff) and Assortativity (AS), across subjects.

## Discussion

In this study, we quantified the convergence of probabilistic structural networks obtained using two independent state-of-the-art reconstruction pipelines over a range of network density thresholds, by merging alternative parcellation schemes to an anatomically equivalent and common node scale. We also replicated our experiment using both the native parcellation scales and an alternative (AAL) parcellation scheme. Our results show there is high agreement between the two alternative reconstruction methods. We observed a global peak convergence corresponding to the brain network that occured most robustly between the two methods. The graph theoretical properties of these ‘consensus networks’ were highly similar between pipelines employing the same atlas but showed some variation across atlases at different node scales. Fibers representing these networks recovered the majority of major white matter tracts in all atlases, giving us confidence that the network has reasonably high anatomical validity.

### Convergence between alternative network reconstructions

Individual components of the structural network reconstruction pipeline can impact on network anatomical accuracy or the network metrics, meaning the combinatorial choice of which complete reconstruction pipeline to employ is of great importance. Previous studies have found that graph theoretical properties of binary structural brain networks, such as hierarchical modularity and small-worldness, were similar across alternative acquisition and parcellation methods (at the same node scale) [57], as well as between alternative connection weighting schemes [31], [58]. However, the agreement between completely independent reconstruction methods of similar capability has not been previously addressed. We found that the mean DC across all subjects and densities was significantly higher than by chance for all atlases, meaning that alternative reconstructions yielded connection weights that were ranked similarly across the entire rank profile. We therefore observed a highly significant agreement between structural networks obtained from two independent state-of-the-art reconstruction pipelines for the first time. This is an important finding since it demonstrates agreement between individual network connections as opposed to network topological measures, which may have resulted from a wider array of connection configurations. Furthermore, we can have some confidence that the networks are robust to swapping individual stages between the pipelines to some degree.

A high similarity may be expected given the similar capabilities of the pipelines. High correlation in connection rank profiles is an intrinsic property of probabilistic tractography studies, whereby probabilistic fibers tend to disperse from the true anatomical tracts as they encounter complex fiber architectures or noise in the diffusion data. This leads to densely populated network weights where the connectivity profile of neighbouring nodes is highly correlated. This may be the primary reason for such a high convergence across the entire density range, even at very low density thresholds (Fig. 4). Although highly significant relative to random networks, the similarity of AAL atlas pipelines was lower than the other atlases. This was due to a number of subjects' weighted networks having a relatively high number of non-connected (and therefore randomly ranked) region pairs, leading to a lower convergence across thresholds (Fig. S1).

A global maximum convergence across thresholds was identified and this corresponded to a sparse network with approximately 100–300 connections (network densities of 0.1–0.2), depending on the atlas. We propose that the connections in the consensus network correspond highly with the underlying anatomical substrate compared to other thresholds. Therefore, for studies employing similar network reconstruction methods, we speculate that this is an appropriate threshold to apply to the weighted networks for balancing sensitivity and specificity to true brain connections.

The convergence decreased sharply towards zero when the network density was below the peak convergent density. This may be explained by factors such as the relatively large impact of rank mismatches in connections between pipelines (due to differences in their respective sensitivity) when the number of connetions is low, or a homogenous weight distribution of the highest ranking connections leading to effectively random ranking and lower convergence.

Reus *et. al.* (2013) [30] recently assessed the impact of threshold on the sensitivity and specificity of brain network connections. Using the Desikan-Killiany atlas with 68 nodes, they estimated the number of true positive connections as 420.7 (corresponding to a network density of 18.5%), which is slightly higher than our study. This difference could be explained by their use of a different experimental design, whereby a model of the true positive distribution was fitted to the prevalance distribution calculated across subject binary networks obtained from deterministic tractography. It is interesting to note that while [30] and our study used different pipelines and analysis methods, the estimate of the number of brain connections is of a similar magnitude. The number of connections in the consensus networks was 110, 152 and 242 (corresponding to network densities of 0.196, 0.161 and 0.106) for atlas of node scale 34, 44 and 68, respectively. This trend of an increasing number of connections occuring consistently between pipelines at higher node scales may be expected given the increase in possible region pairs. Similarly, [30] found that the estimated number of true positive connections increased in the Harvard-Oxford atlas, which has 96 nodes.

Other studies have investigated the effect of threshold on anatomical validity of structural networks, by utilising a ground truth for particular sub-components of the network. Li *et. al.* (2012) investigated the effect of threshold on the sensitivity and specificity of structural network connections, using connectvity data derived from post-mortem tract tracing techniques in the macaque brain as a ground truth [31]. The performance of several tractography strategies was assessed by analysing the area under the receiver operating characteristic curve. However, their study was limited to a subset of brain regions due to limited availability of tract tracing data in macaque brain and an optimal threshold for performing network analysis was not reported. Bastiani *et. al.* (2012) used a network ‘quality control’ technique to analyse the sensitivity and specificity of brain network connections across thresholds for deterministic tractography reconstruction techniques [59]. However, their sensitivity and specificity metrics measured connectivity between sets of regions known to be connected according to *a priori* information and therefore may have had limited applicability to other connections across the network. In contrast to these works, our study utilised information across all brain region pairs and compared two independent pipelines as opposed to performing a more focussed study on individual fiber tractography reconstruction stages.

We found high similarity in graph theoretical propeties of the consensus networks when comparing those derived from the same node scale atlas. This may be expected, given the convergence of connections at the peak convergence density was above 65%, and that the networks had the same connection density, which is known to significantly impact upon the graph theoretical metrics [32]. Despite high similarity across alternative pipelines at the same node scale, some graph theoretical properties were significantly different across node scales. Most notably, the number of connections in the consensus network increased and density of the network decreased in atlases at higher node scales. This could be due to division of connections between multiple parcels at higher node scales due to an increase in the number of possible regional pairs.

We found that the peak convergence density using the AAL atlas was similar to that of the Desikan-Killiany atlas, despite the AAL atlas having a higher node scale. This may be because the AAL atlas uses a fundamentally different type of parcellation scheme and algorithm. While the Desikan-Killiany atlas contains only the grey matter region of the cortex, the AAL atlas represents larger regions which include both grey and white matter. This may have increased the number of robustly occurring cortical connections, as some streamlines, which may otherwise have become truncated before reaching the grey matter of the cortex (due to noise and tissue partial volume effects), intersect these parcels. Furthermore, the AAL segmentation algorithm uses an affine registration between the subject brain and a standard brain from MNI space, meaning subject differences in brain morphology are not considered. Larger parcels and limited ability to account for individual brain variation may have meant single connections became distributed across multiple parcels in the network, leading to a high peak convergence network density compared to the Hammers and Desikan-Killiany atlases (Fig. 4).

With the exception of the AAL atlas, the pathlength and global efficiency tended to increase and decrease, respectively, in consensus networks with increasing node scale atlases. This may due to the lower consensus network densities at higher node scales, resulting in a decrease in the ratio of edges to nodes. Also, clustering coefficient and local efficiency decreased when comparing the consensus networks obtained from the Hammers (44 nodes) to the Desikan-Killiany atlas (68 nodes). Zalesky *et. al.* (2010) examined the effect of node scale on the pathlength and clustering coefficient of networks generated using deterministic tractography and found that an increase and decrease in the pathlength and clustering coefficient metrics, respectively [60]. Their study examined a wide range of node scales (from 82 to 4000 in steps of 500) whereas our study re-affirms these findings at finer node scale increments. However, clustering and local efficiency showed no clear trend with node scale when comparing the Common (34 nodes) and Hammers atlases (44 nodes).

It should be noted that although the observed correspondence of connection fiber paths with known white matter tracts does suggests some degree of anatomical truth to the underlying connections in the consensus networks (Fig. 5), spurious network weights may be included in the consensus networks due to a common bias in the tractography methods. Therefore, although the reconstructed tracts are sensible, they are unlikely to be exhaustive. For example, local tractography techniques may produce shorter fibers than found *in vivo*, as fibers are deflected from the true path due to noise and limited angular resolution. This may have meant connection weights between distant regions were lower than expected. Some network reconstruction methods have accounted for this by penalizing the weighting of shorter inter-regional distances [31]. In addition to the weighting scheme, many other alternative pipelines are available which may result in different convergence results. Therefore, it should be emphasised that we demonstrated agreement between two pipelines out of a large number of possibilities and that our results may not apply to pipelines which employ different parcellation, registration or tractography methods. Finally, the peak convergent threshold described here was derived from a population of healthy individuals and may not represent an appropriate threshold for other clinical populations where connections may have become altered or absent.

## Conclusion

High convergence between two independent state-of-the-art structural network reconstruction pipelines was observed on a connection-wise basis for all density thresholds. A sparse ‘consensus network’, which occured most robustly between the pipelines, was identified in four atlases, and had a density of between 10% and 20% (100–250 connections). We propose that these connections have high anatomical validity compared to other thresholds, which is useful given the inherent difficulty in defining thresholds for brain network studies. The pipeline had relatively little effect on the network properties of the consensus networks, although some relationship with atlas node scale was observed, in agreement with previous studies. When performing structural network analysis, it may be useful to apply multiple pipelines to diffusion-weighted data and to use the comparison framework described here to identify the most important connections.

## Supporting Information

Figure S1
**Normalised dice similarity coefficient between pipelines across density thresholds for all subjects.** The normalised similarity coefficient was calculated by subtracting the expected from the observed dice coefficient at each density. Shown are the the subject normalised dice coefficients (left) and the mean normalised dice coefficient 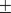 standard deviation (right) for pipelines using the Common (top), Hammers (middle-top), Desikan-Killiany (middle-lower) and AAL (lower) atlases. A peak normalised dice coefficient is observed for densities in the region of 0.05–0.20 for all atlases.(TIF)
