# Supplementary figures and images for: Consensus between Pipelines in Structural Brain Networks
Source: PLoS One. 2014 Oct 30;9(10):e111262. doi: 10.1371/journal.pone.0111262 (PMC4214749; doi:10.1371/journal.pone.0111262)

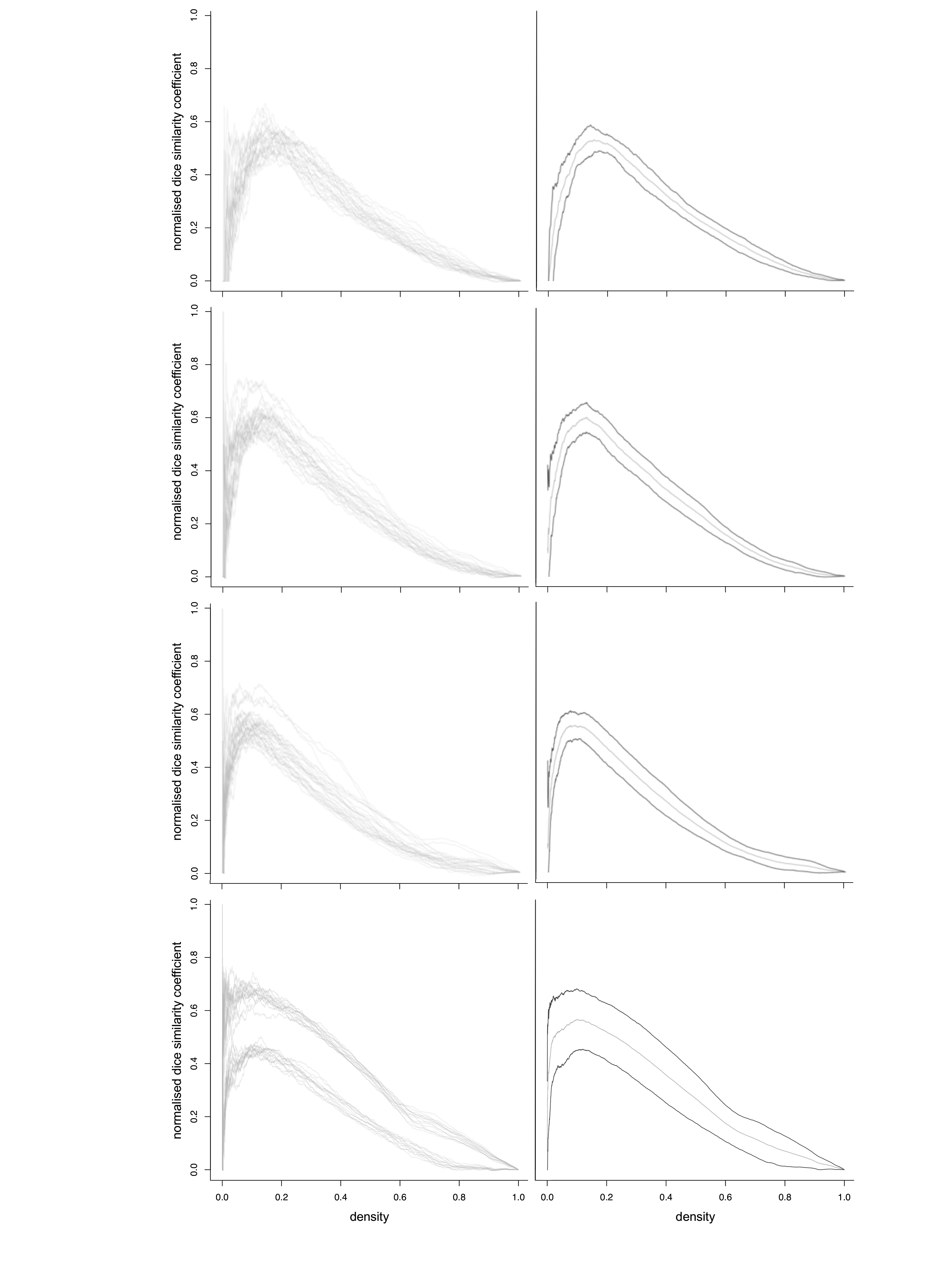

Supplement: Figure S1 — Normalised dice similarity coefficient between pipelines across density thresholds for all subjects. The normalised similarity coefficient was calculated by subtracting the expected from the observed dice coefficient at each density. Shown are the the subject normalised dice coefficients (left) and the mean normalised dice coefficient standard deviation (right) for pipelines using the Common (top), Hammers (middle-top), Desikan-Killiany (middle-lower) and AAL (lower) atlases. A peak normalised dice coefficient is observed for densities in the region of 0.05–0.20 for all atlases. (TIF) [file pone.0111262.s001.tif]
